# Supplementary material for: Content-rich biological network constructed by mining PubMed abstracts
Source: BMC Bioinformatics. 2004 Oct 8;5:147. doi: 10.1186/1471-2105-5-147 (PMC528731; doi:10.1186/1471-2105-5-147)
Supplement: Additional File 2 — The original results of the above study (non-essential files are deleted to keep the file size under the limit set by BMC bioinformatics). [file 1471-2105-5-147-S2.bz2 › chilibotAdditionalFile2/dip50/665ID10428814E11059/html/TNFRSF5_TRAF3.html]

 


 **TNFRSF5** and **TRAF3** 
  
Found 115 abstracts in PubMed, retrieved 50.  
 

 What does Google say? 
 PDF only 
| .edu only 

---

**Interactive relationship** (e.g. stimulation, inhibition, etc)

**Inhibitory relationship**- Upregulation of TRAF 3  [ **TRAF3** ]  by shear stress blocks CD40  [ **TNFRSF5** ]  mediated endothelial activation.  Ref: 11714736 J Clin Invest, 2001
- Humoral immune responses of LMP1  [ **TRAF3** ]  transgenic mice in CD40  [ **TNFRSF5** ]  deficient or normal backgrounds revealed that LMP1  [ **TRAF3** ]  mimics CD40  [ **TNFRSF5** ]  signals to induce extrafollicular B cell differentiation but, unlike CD40  [ **TNFRSF5** ] , blocks germinal center formation.  Ref: 10514374 Science, 1999
- Antisense oligodeoxynucleotide ODN for  **TRAF3**  inhibited CD40  [ **TNFRSF5** ]  induced activation of MEK1 ERK pathway by decreasing expression of  **TRAF3**  protein, but antisense ODNs for TRAF, and 6 were ineffective.  Ref: 12220533 Arch Biochem Biophys, 2002
- Transiently expressed soluble trimeric CD40  [ **TNFRSF5** ]  cytoplasmic domain was isolated as complexes that contained TRAF2,  **TRAF3** , TRAF5, TRAF6, and the inhibitor of apoptosis protein c IAP1.  Ref: 11562359 J Biol Chem, 2001
- Although B95.8 LMP1  [ **TRAF3** ]  induced growth inhibition, expression of certain cell surface molecules CD40  [ **TNFRSF5** ] , CD44, and CD54, and secretion of interleukin 6 and 8 in SCC12F cells, stable CAO LMP1  [ **TRAF3** ]  expression failed to elicit these effects.  Ref: 10873763 Virology, 2000
- Furthermore, CD40  [ **TNFRSF5** ]  mediated enhancement of IL 4 driven germline C epsilon transcription was inhibited by antisense ODN for  **TRAF3**  and by a MEK1 inhibitor, PD98059.  Ref: 12220533 Arch Biochem Biophys, 2002
**Stimulatory relationship**- ASM, ceramide, and CD40  [ **TNFRSF5** ]  colocalize in the cap  [ **TRAF3** ]  like structure of stimulated cells.  Ref: 11751974 J Immunol, 2002
- CD40  [ **TNFRSF5** ]  mutants with impaired binding to TRAF2,  **TRAF3** , or both of these proteins completely retained the ability to activate NF kappaB and Jun N terminal kinase JNK, implying that CD40  [ **TNFRSF5** ]  can stimulate TRAF2 and  **TRAF3**  independent pathways for NF kappaB and JNK activation.  Ref: 10428814 J Biol Chem, 1999
- In a 293 cell line stably expressing CD40  [ **TNFRSF5** ] ,  **TRAF3**  is released from the  **TRAF3** .MIP T3 complex and recruited to the CD40  [ **TNFRSF5** ]  receptor upon CD40  [ **TNFRSF5** ]  ligand stimulation.  Ref: 10791955 J Biol Chem, 2000
- In these cases the Hodgkin and Reed Sternberg HRS cells express the EBV encoded proteins LMP1  [ **TRAF3** ]  and LMP2, which can mimic CD40  [ **TNFRSF5** ]  and the B cell receptor, respectively, and therefore may play a critical role in facilitating the survival of HRS cells.  Ref: 12078898 Ann Oncol, 2002
- Using CD40  [ **TNFRSF5** ]  mutants with impairments in their ability to bind selected TNF receptor associated factor family proteins, we observed that CD40  [ **TNFRSF5** ]  mediated transcriptional induction of the germ line Ig Cgamma1 and Ig Cepsilon promoters was markedly reduced by mutations that prevent TRAF2,  **TRAF3** , TRAF5 or TRAF6 binding.  Ref: 10601998 Eur J Immunol, 1999
**Neutral relationship**- Molecular basis for CD40  [ **TNFRSF5** ]  signaling mediated by  **TRAF3** .  Ref: 10984535 Proc Natl Acad Sci U S A, 2000
- Downstream regulator TANK binds to the CD40  [ **TNFRSF5** ]  recognition site on  **TRAF3** .  Ref: 12005438 Structure Camb , 2002
- This suggests that LMP1  [ **TRAF3** ]  utilizes a modified CD40  [ **TNFRSF5** ]  signaling pathway.  Ref: 11304555 J Exp Med, 2001
- TANK and CD40  [ **TNFRSF5** ]  bind to the same crevice on  **TRAF3** .  Ref: 12005438 Structure Camb , 2002
- LMP 1  [ **TRAF3** ]  also differs from CD40  [ **TNFRSF5** ]  in its apparent independence of a ligand for its signaling.  Ref: 12388716 J Virol, 2002
- The stability of  **TRAF3**  regulates CD40  [ **TNFRSF5** ]  NF kappa B mediated control of the immune response, which is central to the biologic activity of the Reed Sternberg cell.  Ref: 11023520 Blood, 2000
- LMP 1  [ **TRAF3** ]  differs dramatically in its structure from CD40  [ **TNFRSF5** ] .  Ref: 12388716 J Virol, 2002
- CD40  [ **TNFRSF5** ]  ligation downregulates EBNA 2 and LMP 1  [ **TRAF3** ]  expression in EBV transformed lymphoblastoid cell lines.  Ref: 12115504 Int J Cancer, 2002
- Here we have defined the oligomeric structure of recombinant TRAF domains that directly interact with CD40  [ **TNFRSF5** ]  and quantitated the affinities of TRAF2 and  **TRAF3**  for CD40  [ **TNFRSF5** ] .  Ref: 10433725 Biochemistry, 1999
- LMP 1  [ **TRAF3** ]  has six membrane spanning domains as opposed to CD40  [ **TNFRSF5** ]  s one.  Ref: 12388716 J Virol, 2002
- In surface plasmon resonance studies, a hierarchy of affinity of trimeric TRAFs for trimeric CD40  [ **TNFRSF5** ]  was found to be TRAF2 >  **TRAF3**  >> TRAF1 and TRAF6.  Ref: 10433725 Biochemistry, 1999
- CD40  [ **TNFRSF5** ]  and LMP1  [ **TRAF3** ]  bind similar sets of cellular signalling proteins and activate overlapping signalling pathways.  Ref: 12401515 Cell Signal, 2003
- The structural moiety of LMP 1  [ **TRAF3** ]  that distinguishes it from CD40  [ **TNFRSF5** ]  and TNF R1, its amino terminus and multiple membrane spanning segments, alone can mediate its cytostatic activity.  Ref: 10723131 Oncogene, 2000
- Fusing LMP 1  [ **TRAF3** ]  s N terminus and membrane spanning domains to CD40  [ **TNFRSF5** ]  s C terminus supports signaling more efficiently than CD40  [ **TNFRSF5** ]  plus ligand or CD40  [ **TNFRSF5** ]  s trimerized and or localized derivatives.  Ref: 11387199 EMBO J, 2001
- We demonstrate that activated CD40  [ **TNFRSF5** ]  and LMP 1  [ **TRAF3** ]  co localize in lipid rafts and recruit  **TRAF3**  there, findings consistent with signals of CD40  [ **TNFRSF5** ]  and LMP 1  [ **TRAF3** ]  being initiated from lipid rafts.  Ref: 11387199 EMBO J, 2001
- We found that CD40  [ **TNFRSF5** ]  engagement also results in its recruitment, together with TRAF2 and  **TRAF3** , to membrane microdomains, regions of the plasma membrane enriched in signaling molecules such as the Src family kinases.  Ref: 10748139 J Biol Chem, 2000
- The data presented here show that the shared binding site for TRAF2 and  **TRAF3**  accounts for receptor internalization, and the majority of signaling through CD40  [ **TNFRSF5** ] , but is redundant with the TRAF6 binding site for activation of p38 and NFkappaB signaling pathways.  Ref: 11754002 Eur J Immunol, 2002
- The latent membrane protein 1 LMP 1  [ **TRAF3** ]  of Epstein Barr virus EBV functionally resembles a constitutively active, CD40  [ **TNFRSF5** ]  like receptor and contributes to the maintenance of proliferation of EBV infected primary human B lymphocytes.  Ref: 11000250 J Virol, 2000
- Latent membrane protein 1 LMP1  [ **TRAF3** ]  is an Epstein Barr virus EBV encoded, ligand independent receptor that mimics CD40  [ **TNFRSF5** ] .  Ref: 12805217 EMBO J, 2003
- Latent membrane protein 1 LMP1  [ **TRAF3** ] , a protein encoded by Epstein Barr Virus EBV, appears to mimic CD40  [ **TNFRSF5** ]  in multiple ways.  Ref: 12401515 Cell Signal, 2003
- CD40  [ **TNFRSF5** ]  crosslinking led to a partial downregulation of EBNA 2, EBNA3 6 and LMP 1  [ **TRAF3** ]  in LCLs, paralleled by downregulation of Cp promoter activity.  Ref: 12115504 Int J Cancer, 2002
- CD40  [ **TNFRSF5** ] , a member of the TNFR 1 receptor family, shares several features with LMP 1  [ **TRAF3** ] , an oncoprotein encoded by Epstein Barr virus.  Ref: 11387199 EMBO J, 2001
- The tumor necrosis factor receptor associated factor 1 TRAF1 participates in the signal transduction of various members of the tumor necrosis factor receptor TNFR family, including TNFR2, CD40  [ **TNFRSF5** ] , CD30, and the Epstein Barr virus EBV encoded latent membrane protein 1 LMP1  [ **TRAF3** ] .  Ref: 11567226 Hum Pathol, 2001
- The tumour necrosis factor receptor associated factors TRAFs 1 and 2 participate in the signal transduction of various members of the tumour necrosis factor receptor TNFR family, including TNFR1, TNFR2, CD40  [ **TNFRSF5** ] , CD30, and the Epstein Barr virus EBV encoded latent membrane protein 1 LMP1  [ **TRAF3** ] .  Ref: 11400143 J Pathol, 2001
- Latent membrane protein 1 LMP1  [ **TRAF3** ]  plays a critical role in B cell transformation by Epstein Barr virus EBV and appears to mimic a constitutively active CD40  [ **TNFRSF5** ]  receptor.  Ref: 11304555 J Exp Med, 2001
- The LMP 1  [ **TRAF3** ]  oncoprotein of EBV is required to maintain proliferation of infected B cells and shares several features with CD40  [ **TNFRSF5** ] , TNF R1, and related receptors.  Ref: 10723131 Oncogene, 2000
- Latent membrane protein 1 LMP1  [ **TRAF3** ]  is an EBV encoded transforming protein that strongly mimics the B cell activating properties of a normal cellular membrane protein, CD40  [ **TNFRSF5** ] .  Ref: 11698454 J Immunol, 2001
- Using a model system designed to directly compare the signaling potency of the cytoplasmic domains of LMP1  [ **TRAF3** ]  and CD40  [ **TNFRSF5** ]  in B lymphocytes, we found that LMP1  [ **TRAF3** ]  more potently activates c Jun kinase and nuclear factor kappaB and induces higher levels of several B cell effector functions than does CD40  [ **TNFRSF5** ] .  Ref: 11304555 J Exp Med, 2001
- The TRAF 3  [ **TRAF3** ]  gene encodes a number of splice variant isoforms that function as adapter molecules in NF kappaB signaling, in part by associating with the cytoplasmic tails of CD40  [ **TNFRSF5** ]  or other TNF receptor TNF R family members.  Ref: 10781837 Mol Immunol< MedlineTA>Mol Immunol, 2001

**Non-interactive relationship** (e.g. studied together, co-existance, homology, etc.)

- While CD40  [ **TNFRSF5** ]  and TNF R1 are dependent on binding their ligands for their signaling, LMP 1  [ **TRAF3** ]  apparently is not.  Ref: 10723131 Oncogene, 2000
- CD40  [ **TNFRSF5** ]  and LMP 1  [ **TRAF3** ]  activate transcription by binding to TRAFs, JAK3 and or TRADD.  Ref: 11387199 EMBO J, 2001
- We report here that CD40  [ **TNFRSF5** ]  induced  **TRAF3**  dependent activation of mitogen activated protein kinase MAPK extracellular signal regulated kinase ERK kinase 1 MEK1 is involved in the upregulation of IL 4 driven germline C epsilon transcription in a human Burkitt s lymphoma B cell line, DG75.  Ref: 12220533 Arch Biochem Biophys, 2002
- A carboxyl truncation mutant of CD40  [ **TNFRSF5** ]  lacking the last 32 amino acids required for TRAF2 and  **TRAF3**  binding, CD40  [ **TNFRSF5** ]  Delta32, mediated NF kappaB induction through a mechanism that was suppressible by co expression of TRAF6 DeltaN, a dominant negative version of TRAF6, but not by TRAF2 DeltaN, implying that while TRAF6 does not directly bind CD40  [ **TNFRSF5** ] , it can participate in CD40  [ **TNFRSF5** ]  signaling.  Ref: 10428814 J Biol Chem, 1999
- LMP1  [ **TRAF3** ]  and CD40  [ **TNFRSF5** ]  both associate with the cytoplasmic adapter proteins called TNFR associated factors TRAFs.  Ref: 11698454 J Immunol, 2001
- we demonstrate that a fusion of LMP1  [ **TRAF3** ]  with CD40  [ **TNFRSF5** ]  recruits TRAF2 intracellularly.  Ref: 12805217 EMBO J, 2003
- Intracellular tumor necrosis factor TNF receptor associated factor TRAF adapter proteins, shown to contribute to signaling by both CD40  [ **TNFRSF5** ]  and LMP1  [ **TRAF3** ] , were recruited by both molecules to lipid enriched membrane rafts.  Ref: 11304555 J Exp Med, 2001
- The latent membrane protein 1 LMP 1  [ **TRAF3** ]  oncoprotein of Epstein Barr virus EBV is a constitutively active, CD40  [ **TNFRSF5** ]  like cell surface signaling protein essential for EBV mediated human B cell immortalization.  Ref: 12610149 J Virol, 2003
- We modified the phenotype of the Group I BL cells by LMP1  [ **TRAF3** ]  transfection or CD40  [ **TNFRSF5** ]  ligation.  Ref: 12115526 Int J Cancer, 2002
- In contrast, LMP1  [ **TRAF3** ]  activated CD40  [ **TNFRSF5** ]  or CD54 ICAM1 expression in the analyzed cell lines.  Ref: 11162833 Virology, 2001
- The crystal structure of a region of TANK bound to  **TRAF3**  has been determined and compared to a similar CD40  [ **TNFRSF5** ]   **TRAF3**  complex.  Ref: 12005438 Structure Camb , 2002
- The fusion of the transmembrane domain of LMP1  [ **TRAF3** ]  to signaling domains of CD40  [ **TNFRSF5** ] , TNF R1 and Fas activates their signaling.  Ref: 12805217 EMBO J, 2003
- We have determined the crystal structure of a 20 residue fragment from the cytoplasmic domain of CD40  [ **TNFRSF5** ]  in complex with the TRAF domain of  **TRAF3** .  Ref: 10984535 Proc Natl Acad Sci U S A, 2000
- In this review, we will compare and contrast the signalling mediated by CD40  [ **TNFRSF5** ]  and LMP1  [ **TRAF3** ] .  Ref: 12401515 Cell Signal, 2003
- Mimicry of CD40  [ **TNFRSF5** ]  signals by Epstein Barr virus LMP1  [ **TRAF3** ]  in B lymphocyte responses.  Ref: 10514374 Science, 1999
- CD40  [ **TNFRSF5** ]  and LMP 1  [ **TRAF3** ]  both signal from lipid rafts but LMP 1  [ **TRAF3** ]  assembles a distinct, more efficient signaling complex.  Ref: 11387199 EMBO J, 2001
- However, TRAF5 could be recruited to wild type CD40  [ **TNFRSF5** ]  in a  **TRAF3**  dependent manner but not to a CD40  [ **TNFRSF5** ]  mutant Q263A that selectively fails to bind  **TRAF3** .  Ref: 10428814 J Biol Chem, 1999
- Crystal structures of the TRAF domain of human TRAF2 in complex with peptides from the TNFR family members CD40  [ **TNFRSF5** ] , CD30, Ox4 1BB, and the EBV oncoprotein LMP1  [ **TRAF3** ]  revealed a conserved binding mode.  Ref: 10518213 Mol Cell, 1999
- Comparison of the interactions of CD40  [ **TNFRSF5** ]  with  **TRAF3**  vs. TRAF2 suggests that CD40  [ **TNFRSF5** ]  may assume different conformations when bound to different TRAF family members.  Ref: 10984535 Proc Natl Acad Sci U S A, 2000
- CD40  [ **TNFRSF5** ]  and its viral mimic, LMP1  [ **TRAF3** ]  similar means to different ends.  Ref: 12401515 Cell Signal, 2003
- Despite many similarities shared between CD40  [ **TNFRSF5** ]  and LMP1  [ **TRAF3** ] , they also differ substantively.  Ref: 12401515 Cell Signal, 2003
- LMP1  [ **TRAF3** ] , an LMP1  [ **TRAF3** ]  transmembrane domain chimera with the LTbetaR signaling domain, and LMP1  [ **TRAF3** ]  CD40  [ **TNFRSF5** ]  activate NF kappaB in wt or aly murine embryo fibroblasts.  Ref: 11278268 J Biol Chem, 2001
- Taken together, these data further our understanding of the function of LMP1  [ **TRAF3** ]  in epithelial cells and suggest that LMP1  [ **TRAF3** ] , similar to its mammalian homolog CD40  [ **TNFRSF5** ] , can exert opposing effects on cell survival depending on the nature of the apoptosis trigger.  Ref: 12504573 Virology, 2002
- Stable transfectants expressing wild type or mutant forms of the cytoplasmic domain of CD40  [ **TNFRSF5** ]  suggested that  **TRAF3** , but not TRAF2, binding was essential for CD40  [ **TNFRSF5** ]  mediated RANTES expression.  Ref: 12122011 J Biol Chem, 2002
- LMP 1  [ **TRAF3** ]  is targeted to the plasma membrane, where it binds TRAF, TRADD, and JAK molecules to activate NF kappaB, AP 1, and STAT dependent pathways as does CD40  [ **TNFRSF5** ] .  Ref: 11000250 J Virol, 2000
- Differential signaling and tumor necrosis factor receptor associated factor TRAF degradation mediated by CD40  [ **TNFRSF5** ]  and the Epstein Barr virus oncoprotein latent membrane protein 1 LMP1  [ **TRAF3** ] .  Ref: 11304555 J Exp Med, 2001
- Additionally, fusion constructs of the transmembrane domain of LMP1  [ **TRAF3** ]  and the intracellular signaling domain of CD40  [ **TNFRSF5** ] , TNF R1, and TNF R2 likewise transactivated the CD83 promoter via NF kappaB.  Ref: 12857898 J VirolJ Virol, 2001
- These results suggest that in DG75 cells,  **TRAF3**  induced MEK1 activation may be involved in CD40  [ **TNFRSF5** ]  mediated upregulation of IL 4 driven germline C epsilon transcription.  Ref: 12220533 Arch Biochem Biophys, 2002
- The binding site for TRAF2 and  **TRAF3**  but not for TRAF6 is essential for CD40  [ **TNFRSF5** ]  mediated immunoglobulin class switching.  Ref: 12354380 Immunity, 2002
- In contrast, LMP1  [ **TRAF3** ]  CD40  [ **TNFRSF5** ]  is inhibited by both wild type wt and aly DN NIK.  Ref: 11278268 J Biol Chem, 2001
- Using a Hodgkin cell line, this study demonstrates that CD40  [ **TNFRSF5** ]  activation of NF kappa B is mediated by proteolysis of  **TRAF3** .  Ref: 11023520 Blood, 2000
- The functional effects of over expressing a TRAF 3  [ **TRAF3** ]  binding fragment, p62 aa 336 522 were studied on NF kappaB dependent, or control STAT1 dependent reporter activity in 293 T cells, either resting or after stimulation by CD40  [ **TNFRSF5** ]  or IFN gamma, respectively.  Ref: 10781837 Mol Immunol< MedlineTA>Mol Immunol, 2000
- Since CD40  [ **TNFRSF5** ]  crosslinking can reportedly inhibit the growth of EBV transformed lymphoblastoid cell lines LCLs, we have examined the effect of CD40  [ **TNFRSF5** ]  ligation on the expression of EBNAs and LMP 1  [ **TRAF3** ]  and on Cp EBV promoter activity together with several phenotypic markers.  Ref: 12115504 Int J Cancer, 2002
- The effects of resistin on expression of vascular cell adhesion molecule VCAM 1 and monocyte chemoattractant chemokine MCP 1 were studied in addition to CD40  [ **TNFRSF5** ]  receptor, CD40  [ **TNFRSF5** ]  ligand induced MCP 1 expression, and tumor necrosis factor receptor associated factor 3  **TRAF3** , an inhibitor of CD40  [ **TNFRSF5** ]  signaling.  Ref: 12874180 CirculationCirculation, 2002
- In contrast to TRAF2 and  **TRAF3** , direct binding of TRAF1, TRAF4, TRAF5, or TRAF6 to CD40  [ **TNFRSF5** ]  was not detected.  Ref: 10428814 J Biol Chem, 1999
- In this study, using isothermal titration calorimetry ITC, we report thermodynamic characterization of the interaction between TRAF2 and monomeric peptide sequences from TNFR members, including TNFR2, CD40  [ **TNFRSF5** ] , CD30, Ox40, and 4 1BB, and the Epstein Barr virus EBV transforming protein, latent infection membrane protein 1 LMP1  [ **TRAF3** ] .  Ref: 10908665 Proc Natl Acad Sci U S A, 2000
- However, we found that TRAFs 2 and 3 were subsequently degraded after CD40  [ **TNFRSF5** ]  but not LMP1  [ **TRAF3** ]  induced signaling.  Ref: 11304555 J Exp Med, 2001
- These results suggest that binding to TRAF2 and or  **TRAF3**  but not TRAF6 is essential for CD40  [ **TNFRSF5** ]  isotype switching and activation in B cells.  Ref: 12354380 Immunity, 2002
- Furthermore, resistin upregulates adhesion molecules and chemokines and downregulates TRAF 3  [ **TRAF3** ] , an inhibitor of CD40  [ **TNFRSF5** ]  ligand signaling.  Ref: 12874180 CirculationCirculation, 2002
- The recognition motif PxQxT is presented in a boomerang like structure in TANK that is markedly different from the hairpin loop that forms in CD40  [ **TNFRSF5** ]  upon binding to  **TRAF3** .  Ref: 12005438 Structure Camb , 2002
- Here, we demonstrate that shear stress prevents CD40  [ **TNFRSF5** ]  ligand induced endothelial cell activation, and we identify upregulation of TNF receptor associated factor 3  [ **TRAF3** ]  TRAF 3  [ **TRAF3** ]  as a potent CD40  [ **TNFRSF5** ]  inhibitory mechanism.  Ref: 11714736 J Clin Invest, 2001
- To define the role of TRAF proteins in CD40  [ **TNFRSF5** ]  dependent isotype switching in B cells, we introduced wild type WT and mutant CD40  [ **TNFRSF5** ]  transgenes that lacked the binding motifs for TRAF6 CD40deltaTRAF6, TRAF2 and  **TRAF3**  CD40deltaTRAF2 3, or both CD40deltaTRAFs into B cells of CD40  [ **TNFRSF5** ]  mice.  Ref: 12354380 Immunity, 2002
- Like ligand activated CD40  [ **TNFRSF5** ] , LMP 1  [ **TRAF3** ]  activates NF kappaB and Jun kinase signaling pathways via binding, as a constitutive oligomer, to tumor necrosis factor receptor associated factors TRAFs.  Ref: 12610149 J Virol, 2003
- The critical role of TRAF1 in the regulation of TRAF2 dependent JNK signaling is particular to the TRAF binding domain of LMP1  [ **TRAF3** ] , since a homologous region in the cytoplasmic tail of CD40  [ **TNFRSF5** ]  or the TRADD interacting domain of LMP1  [ **TRAF3** ]  signal on the JNK axis independently of TRAF1 status.  Ref: 12502848 J Virol, 2003
